# Levodopa-based device-aided therapies for the treatment of advanced Parkinson’s disease: a social return on investment analysis

**DOI:** 10.3389/fpubh.2024.1351808

**Published:** 2024-06-06

**Authors:** Inez Denham, Roxanne Maurin, Irene Deltetto, Anna Stefanie Mikolaizak, Jenny B. Waern, Colman Taylor

**Affiliations:** ^1^HTANALYSTS Pty Ltd., Sydney, NSW, Australia; ^2^AbbVie Pty Ltd., Sydney, NSW, Australia; ^3^The George Institute for Global Health, Sydney, NSW, Australia

**Keywords:** Parkinson’s disease, social return on investment, levodopa, device-aided therapy, social impact

## Abstract

**Introduction:**

Parkinson’s disease (PD) is an incurable, progressive, neurodegenerative disorder. As PD advances and symptoms progress, patients become increasingly dependent on family and carers. Traditional cost-effectiveness analyses (CEA) only consider patient and payer-related outcomes, failing to acknowledge impacts on families, carers, and broader society. This novel Social Return on Investment (SROI) analysis aimed to evaluate the broader impact created by improving access to levodopa (LD) device-aided therapies (DATs) for people living with advanced PD (aPD) in Australia.

**Methods:**

A forecast SROI analysis over a three-year time horizon was conducted. People living with aPD and their families were recruited for qualitative interviews or a quantitative survey. Secondary research and clinical trial data was used to supplement the primary research. Outcomes were valued and assessed in a SROI value map in Microsoft Excel™. Financial proxies were assigned to each final outcome based on willingness-to-pay, economic valuation, and replacement value. Treatment cost inputs were sourced from Pharmaceutical Benefits Schedule (PBS) and Medicare Benefits Scheme (MBS) published prices.

**Results:**

Twenty-four interviews were conducted, and 55 survey responses were received. For every $1 invested in access to LD-based DATs in Australia, an estimated $1.79 of social value is created. Over 3 years, it was estimated $277.16 million will be invested and $406.77 million of social return will be created. This value is shared between people living with aPD (27%), their partners (22%), children (36%), and the Australian Government (15%). Most of the value created is social and emotional in nature, including reduced worry, increased connection to family and friends, and increased hope for the future.

**Discussion:**

Investment in LD-based DATs is expected to generate a positive social return. Over 50% of the value is created for the partners and children of people living with aPD. This value would not be captured in traditional CEA. The SROI methodology highlights the importance of investing in aPD treatment, capturing the social value created by improved access to LD-based DATs.

## Introduction

Parkinson’s disease (PD) is an incurable neurodegenerative disorder characterised by the progressive loss of dopamine-producing neurons in the brain which impairs an individual’s ability to control and coordinate movement (1). In its early stages, PD presents with three main symptoms: uncontrollable shaking (tremor), slowness of movement (bradykinesia), and muscle stiffness (rigidity). Other symptoms include postural instability, nerve pain, cognitive dysfunction, and mood and sleep disturbances (1).

Oral levodopa (LD) is the mainstay in PD treatment (2, 3) and is commonly prescribed in combination with a dopamine decarboxylase inhibitor (commonly carbidopa or benserazide). It works to replenish and maintain dopamine levels in the brain to reduce PD symptoms (2). Oral levodopa is initially effective at controlling PD symptoms, but effectiveness decreases as the disease progresses. This is due to several reasons, including the short half-life of LD leading to variable plasma concentration, and erratic gastric emptying. Additionally, as PD progresses and more dopaminergic neurons lose the capacity to store dopamine, the therapeutic window during which patients experience adequate symptom control narrows, and patients experience periods of severe symptom onset (referred to as “Off” time). To maintain symptom control, people living with advancing PD often require higher and more frequent doses of oral LD, resulting in an increased risk of medication side effects such as dyskinesia (involuntary, erratic movement of the limbs), an increasingly complex oral dosing regimen and, in turn, a greater medication-related burden (2). As a result, people living with advancing PD often require higher levels of care from their partner and family. This has significant impacts on the quality of life (QoL) of both the person living with PD and their family, leading to an increased physical, mental, social, economic, and emotional burden (4–6).

While there is no universally agreed definition of the term ‘advanced PD (aPD)’, it is generally defined as PD which is poorly controlled by oral LD, often based on ‘5–2-1’ criteria (≥ 5 times daily oral LD use, ≥ 2 daily hours of “Off” time, or ≥ 1 daily hour with troublesome dyskinesia). It is characterised by significantly decreased bilateral mobility, severe motor deficits including tremors and rigidity, increased risk of falls, and cognitive and mental health decline (7). Studies have shown a wide variation in the prevalence of aPD among those with PD, ranging from 10 to 60% depending on the setting (8). Additionally, data suggests up to 40% of people with PD will experience symptoms of advanced disease within 5 years of initiating oral LD (9). As aPD patients experience worsening symptoms, LD device-aided therapies (DATs) may be considered to maintain a consistent LD-plasma concentration, provide better symptom control, and reduce the medication-related burden of PD oral medication regimens (2, 9). LD-based DATs provide a continuous infusion of treatment, resulting in a stable plasma concentration of LD and reduction in aPD symptoms (2, 9, 10).

In Australia, LD/CD intestinal gel (Duodopa®) is the only LD-based DAT currently subsidised by the Pharmaceutical Benefits Scheme (PBS). Initiation of Duodopa® requires a percutaneous endoscopic gastrostomy (PEG) procedure where a jejunostomy tube (J-tube) is permanently placed. The medication is then administered through the PEG/J tube by an external device (referred to as “the pump”) (2, 3). The clinical efficacy and safety of Duodopa® is well established, however, analysis of PBS data suggests only 5% of people living with aPD are treated with Duodopa®. LD-based DAT uptake is currently limited by health system capacity constraints and the requirement for surgical initiation.

A continuous subcutaneous infusion of foslevodopa/foscarbidopa (prodrugs of LD and CD that are converted into their active form in the body) (Vyalev®) is now being investigated in the clinical trial setting (11). Like Duodopa®, Vyalev® will provide continuous drug administration provide stable LD-plasma concentration and thus reduce aPD symptoms. However, unlike Duodopa®, Vyalev® does not require surgical initiation.

Some of the economic impacts of PD have been previously documented in traditional cost-effectiveness analyses (CEA). Such analyses consider the direct and tangible costs experienced by the patient and health system but often fail to capture the indirect burden of disease; that is, the intangible costs, including the impact of disease on families and broader society. In 2014, the annual cost of PD in Australia was estimated to be over $1 billion (12). Direct health system costs contributed to most of this estimation. However, much of this data is now out of date and thus, existing economic assessments of PD likely underestimate its true cost. Further, Australian clinicians have called for patient’s QoL as well as more qualitatively subjective non-motor symptoms to be considered when assessing access to advanced treatment (8). As there is a known impact on the QoL of both the person living with PD and their family (4–6), an assessment including the social, emotional, and intangible consequences should also be conducted to understand the true cost of PD.

Social Return on Investment (SROI) is a principles-based research method used to understand, measure, and report the broader social, economic, and environmental consequences of an intervention (13). SROI analyses rely on extensive and robust stakeholder engagement, including with families, carers, and broader society, to measure change in ways that are relevant to those impacted (13). This process of stakeholder engagement captures and compliments outcomes which are often underrepresented or excluded from traditional CEAs. To date, no SROI analysis of aPD device-aided therapies has been undertaken.

This novel study aimed to undertake a SROI analysis to understand, measure, and report the broader social, economic, and environmental consequences of aPD treatment with LD-based DATs (Duodopa® or Vyalev®).

## Methods

The SROI framework has been described in detail elsewhere (13). Briefly, an SROI involves six key stages: (1) establish the scope and identify stakeholders; (2) map outcomes; (3) evidence outcomes and give them a value; (4) establish the impact; (5) calculate the SROI; and (6) report to stakeholders, use the results, and embed the SROI process.

For this analysis, a forecast-type SROI with a 3-year time horizon was chosen, to limit uncertainty associated with reduced clinical effectiveness over time and to capture the short- and medium-term changes in health and social impacts expected to result from treatment with a LD-based DAT. This time horizon was considered reasonable given that clinical data has demonstrated people receiving treatment with LD-based DATs continue to experience statistically significant outcomes up to 36 months after commencing treatment (14).

### Stakeholder engagement and mapping outcomes

#### Stakeholder groups identification

The stakeholders to be considered were groups that may affect or be affected by improving access to LD-based DATs. Some stakeholders were considered appropriate to be consulted as proxies for other groups included in the analysis (Table 1). Seven stakeholder groups were identified as likely to be materially impacted or able to act as a proxy for other groups: people living with aPD, their partners, their children, neurologists, Parkinson’s nurses, patient advocacy groups, and the Australian Government.

**Table 1:** Preliminary list of stakeholders.

Stakeholder	Included/excluded
People living with aPD	Included
Partners of people living with aPD	Included
Children of people living with aPD	Included
Friends and other family of people living with aPD	Excluded
Employers	Excluded
Advocacy organisations (e.g., Parkinson’s Australia and Shake It Up)	Excluded (consulted as proxy)
Nurses caring for people living with aPD	Excluded (consulted as proxy)
Neurologists treating people living with aPD	Excluded (consulted as proxy)
Hospitals	Excluded
The Australian Government	Included
Wider general population	Excluded

aPD, advanced Parkinson’s disease.

#### Participant recruitment and interviews

Neurologists treating people living with aPD were identified by the study sponsor and contacted via email from May to June 2022. One follow-up email was sent to each clinician and no further contact was attempted after this to avoid potential coercion or pressure from the researchers. Once a clinician indicated their willingness to participate, an introductory meeting was held and the project was outlined in more detail. People living with aPD who were eligible for DATs were identified by their treating clinician. Partners and family members were identified by people living with aPD. Invitations for interviews were sent via email, including the consent form and brief details about the research aims, ethical considerations, and confidentiality. PD advocacy and research organisations throughout Australia, specifically *Parkinson’s Australia* and *Shake It Up*, were also contacted to recruit people living with PD for surveys and interviews regarding their experience. Participation and information flyers for the study were disseminated across respective networks via newsletters and email to inform people of the study being conducted.

It was made clear to all stakeholders that the research was being conducted separately from any ongoing clinical trial (specifically relating to the investigational product Vyalev®) and participation in the study would not impact their relationship with the study sponsor or jeopardise their current or future treatment for PD or any other condition.

Interviews were semi-structured and conducted virtually over Zoom or Microsoft Teams and in some cases over the phone. The aim of the interviews was to understand changes in aPD symptoms that had occurred after commencing treatment with a LD-based DAT and any downstream changes in QoL or daily and leisure activities that arose due to symptom changes.

Interviews were analysed thematically to identify common experiences among stakeholders. Interview transcripts were uploaded to Dovetail (15), coded, and tagged by a single researcher. Codes were reviewed by another researcher. Coded transcripts were used to inform the Theory of Change and determine final outcomes.

Surveys were conducted using Qualtrics (16). The aim of the surveys was to understand the relative importance of individual symptoms and outcomes of treatment. Participants were asked to rank which symptoms were most important for them to control and which outcomes were most important for them to experience. The outcomes to be ranked were based on the findings from the interviews and literature. Ranked outcomes were used to inform the importance of each final outcome and the SROI filters.

### Secondary research

Phase III clinical trial data from the M15-736 trial was used to determine the proportion of people expected to experience a change as a result of treatment with a LD-based DAT (11). The M15-736 trial compared treatment with Vyalev®, a LD-based DAT, to continued treatment with oral LD/CD immediate release tablets. The M15-736 clinical trial was considered the most relevant study to assess the clinical impact of LD-based DATs due to its recency compared to Duodopa® clinical trials, capturing the current standard of care in aPD treatment. Efficacy outcomes were measured using the PDQ-39 and the Parkinson’s Disease Sleep Scale (PDSS). Individual domains from the PDQ-39 were analysed to determine the difference in the proportion of people who experienced an improvement in their PD symptoms after commencing treatment. Additionally, a literature search was conducted to identify patient-reported outcomes considered relevant in people living with PD.

Theory of change (ToC) maps were developed for each stakeholder based on consultation and secondary research. Thematic analysis of interview transcripts and narrative analysis of secondary literature was used to identify recurring themes and understand outcomes of importance to people living with aPD and their families.

### Valuing outcomes and establishing impact

Outcomes were valued and assessed in a SROI value map created in Microsoft Excel, which was an adaptation of the Social Value International Value Map available online (17).

An importance weight was applied to each final outcome, to account for the degree to which the outcome was valued from the perspective of stakeholders, informed by stakeholder surveys. Each outcome was also assigned a financial proxy based on three valuation approaches: willingness-to-pay (the value of an outcome based on how much stakeholders are willing to pay/accept), economic valuation (the financial value representing the actual savings/costs to the stakeholder), or replacement value (the cost of other goods or services which would achieve the same amount of change).

SROI filters including deadweight, attribution, displacement, and drop off were determined via stakeholder consultation and secondary research and then quantified. Specifically, deadweight for each outcome was quantified using a six-point scale from never (0%), very probably not (20%), might (40%), probably (60%), very probably (80%) to certainly (100%). This transformation scale was extracted from a previously assured SROI report (18) and accounted for the amount of change that could have happened without the intervention. Similarly, attribution was measured on a six-point Likert scale which measured the contribution of external factors to the outcome. The scale ranged from 0% (the change was completely the result of the intervention) to 100% (the intervention had nothing to do with the change). Displacement and drop off were determined using data from the M15-736 pivotal clinical trial, including the incremental change between treatments and treatment discontinuation rates (19).

In health economic evaluations, discounting is intended to reflect the difference in how society values future outcomes compared to present outcomes. In Australia, the recommended discount rate is 5% (20). This discount rate was applied to each final outcome. Alternative discount rates of 3.5 and 0% are also recommended and tested in the sensitivity analyses (20).

The true cost of the medicine to the Government (also called the ‘effective price’ or ‘net price’) is commercial-in-confidence thus was not used in this SROI. The published drug cost of Duodopa® was used as an input for the cost of LD-based DATs. This cost was separated into Pharmaceutical Benefits Scheme (PBS) costs (21) (paid by the Australian Government) and the co-payment (paid by the patient). The annual cost of Vyalev® per patient was assumed to be equal to that of Duodopa®. The ‘list price’ of Duodopa® was discounted using an average rebate estimate. The average PBS rebate across all listed medicines was calculated based on publicly available PBS expenditure reports for the financial year 2020–2021 (22). This rebate was applied to the Duodopa® list price to calculate an estimated effective price. The cost of medical services associated with commencing LD-based DATs was also included as an input, based on hospital costs and specialist fees (23, 24).

In order to avoid double counting, it was assumed that other stakeholders (e.g., partners and children of people living with aPD) did not have any monetary or in-kind investment into the treatment, and all financial inputs were incurred by the patient themselves.

The number of people living with aPD who would access treatment with LD-based DATs each year totalled 1,228. This was calculated from the number of people currently living with aPD based on ‘5–2-1’ criteria, analysis of PBS data, and clinician feedback and expertise (Supplementary Table S1).

### Ethical considerations

Ethics approval from Bellberry Human Research Ethics Committee (HREC) was received on the 27th of April 2022 (Application No. 2022–01-082). Additional ethics approval was received from the Gold Coast Hospital and Health Service on the 3rd of August (HREC/2022/QGC/87501) to enrol stakeholders treated within public hospitals.

## Results

A total of 79 participants were recruited from May 2022 to March 2023 for semi-structured interviews (*n* = 24) and surveys (*n* = 55) (Table 2). Survey responses were collected between December 2022 and January 2023.

**Table 2:** Stakeholder engagement summary.

Engagement type	Stakeholder	Number of unique engagements
Interview	People living with aPD	4
	Partners of people living with aPD	4
	Neurologists (*as proxy*)	4
	Nurses (*as proxy*)	2
	Patient advocacy groups (*as proxy*)	2
Survey	People living with aPD	38
	Partners of people living with aPD	17
Follow up interview	People living with aPD	5
	Partners of people living with aPD	1
	Nurses (*as proxy*)	2
Total		**79**

aPD, advanced Parkinson’s disease. The bold value indicates the total value of unique engagements.

Fifteen unique outcomes were included in this SROI. Six outcomes for people living with aPD, five outcomes for partners of people living with aPD, two for children of people living with aPD, and two for the Australian Government (see Table 3).

**Table 3:** Included final outcomes.

Stakeholder	Outcome(s)	Outcome type	Rationale
People living with aPD	Reduced out-of-pocket costs for aids and modifications	Economics	“[Oral medication] just did not work for me at all. So, I had 3 months in which I had a number of falls, and I damaged my shoulder quite badly… and then when I went on the [LD-based DAT] infusions, things changed rapidly” – Person living with Parkinson’s disease receiving treatment with a LD-based DAT
	Increased independence	Role functioning	“I spend approximately 65% of my waking day in the “Off” state when my medication is not working. This causes me to have difficulty moving independently, feeding myself, and performing basic tasks. The 35% I manage in the “On” state is with troublesome dyskinesia, very violent movements that again prevent me from doing most basic activities” – Person living with aPD discussing the burden of disease progression (25)
	Increased connection to family and friends	Social and emotional	“The best part is we have a social life again! Reconnecting with my friends and spending time with my family has brought me so much joy and happiness.” – Person living with aPD receiving treatment with a LD-based DAT (25)
	Increased ability to remain in the workforce	Productivity	“My primary goal is to stay at work and retire when I want to retire, not when Parkinson’s makes me retire…” – Person living with aPD receiving treatment with a DAT
	Increased hope for the future	Social and emotional	“[Access to treatment with a LD-based DAT] has improved my life enormously compared to what it was like on the tablets… I just do not have the down times anymore.” – Person living with aPD receiving treatment with a LD-based DAT
	Increased burden of discomfort	Social and emotional	“Having a tube and pump took some time getting used to, but the independence is worth it…” – Person living with aPD receiving treatment with a LD-based DAT (25)
Partners of people living with aPD	Increased hope for the future	Social and emotional	“[Treatment with LD-based DATs] straightens out your life a little bit more. It gives you a bit more hope for the future.” – Partner of patient with aPD receiving treatment with a LD-based DAT
	Reduced worry about partner’s health	Social and emotional	“We are so much happier. We were given life back. My wife does not have to worry anymore.” – Person living with aPD receiving treatment with a LD-based DAT (25)
	Increased carer wellbeing	Wellbeing	“I used to ask him if he could just give me some respite because there was only me” – Partner of person living with PD
	Increased connection to family and friends	Social and emotional	“I could go for walks with my husband, go to the movies, go back to work” – Partner of person living with aPD receiving treatment with a LD-based DAT (25)
	Increased ability to remain in the workforce	Productivity	
Children of people living with aPD	Increased connection to parent	Social and emotional	“[The voice of the person living with aPD had] become very weak and he really could not have phone conversations with his sons who both live interstate. That had become a real issue because he felt he was losing touch… [After starting treatment with a LD-based DAT] his sons were really blown away by having these great long conversations with their dad.” – Nurse caring for people living with aPD receiving treatment with LD-based DATs
	Reduced worry about parent	Social and emotional	“They told us to go to a retirement village” – Person living with aPD talking about their children
Australian Government	Avoided cost of healthcare services	Economics	
	Avoided cost of welfare services and support payments	Economics	

aPD, advanced Parkinson’s disease; DATs, device-aided therapies; LD, levodopa.

The financial proxies for the included outcomes are shown in Table 4. Five financial proxies were based on willingness-to-pay/accept, six on economic valuation, and four on replacement valuation.

**Table 4:** Financial proxy allocated to each outcome.

Stakeholder	Outcome(s)	Valuation approach	Financial proxy	Financial proxy (value)
People living with aPD	Reduced out-of-pocket costs for aids and modifications	Economic valuation	Avoided out-of-pocket cost for aids and modifications	$6,399.81
	Increased independence	Replacement valuation	Level 4 HCP	$53,268.10
	Increased connection to family and friends	Willingness-to-pay/accept	Average weekly household spending	$9,625.48
	Increased ability to remain in the workforce	Economic valuation	Median annual wage based on part-time equivalence	$42,354.00
	Increased hope for the future	Willingness-to-pay/accept	Average annual cost of family holiday	$5,702.00
	Increased burden of discomfort	Willingness-to-pay/accept	Average household spending on clothing and footwear	-$2,186.02
Partners of people living with aPD	Increased hope for the future	Willingness-to-pay/accept	Average annual cost of family holiday	$5,702.00
	Reduced worry about partner’s health	Replacement valuation	Average aged care home costs and fees in Australia	$48,535.77
	Increased carer wellbeing	Replacement valuation	Cost of support worker for 63 days	$22,330.35
	Increased connection to family and friends	Willingness-to-pay/accept	Average weekly household spending	$9,625.48
	Increased ability to remain in the workforce	Economic valuation	Median annual wage based on full-time equivalence	$84,708.00
Children of people living with aPD	Increased connection to parent	Economic valuation	Average cost of a monthly phone-on-a-plan mobile phone contract in Australia	$972.00
	Reduced worry about parent	Replacement valuation	Average aged care home costs and fees in Australia	$48,535.77
Australian Government	Avoided cost of healthcare services	Economic valuation	Avoided cost of healthcare services and utilisation	$26,102,780.40
	Avoided cost of welfare services and support payments	Economic valuation	Avoided cost of welfare services and support payments	$4,464,415.61

aPD, advanced Parkinson’s disease; HCP, Home Care Package.

SROI filters (deadweight, attribution, displacement, and drop off) and importance weights are included in the Supplementary Table S2. By convention, outcomes which are already financial in nature were assigned an importance weighting of 100%.

The total investment into LD-based DATs over the three-year time horizon was calculated to be $227.16 million, or $79.44 million per year. Over the 3-year time horizon, it was estimated $277.16 million will be invested and $406.77 million of social value will be created.

The SROI ratio was estimated to be 1:1.79. This indicates that for every AUD$1 invested to improve access to LD-based DATs in Australia, an estimated AUD$1.79 of social value is created. This value (to be understood as the return on investment) is shared between people living with aPD (27%), their partners (22%), children (36%), and the Australian Government (15%). Most of the value is created from social and emotional outcomes (AUD$221.34 million, 54%), followed by role functioning (AUD$75.02 million, 18%), economics (AUD$62.92 million, 15%), productivity (AUD$27.44 million, 7%), and wellbeing (AUD$20.06 million, 5%).

Sensitivity analyses of recommended discount rates, cost inputs, valuation approaches, time horizon and duration, and SROI filters showed all alternative scenarios yield a positive SROI (range 1:1.07 to 1:2.24) (Supplementary Table S3).

The analysis identified an increased burden of discomfort which was associated with the external pump required for both Duodopa® and Vyalev®. However, PD nurses noted that this burden could often be avoided if patients receive adequate support and education prior to commencing treatment with a LD-based DAT. PD nurses help to support patients with questions about their disease and can provide education regarding treatment optimisation. The lack of access to specialised PD nurses was noted as a barrier to uptake of LD-based DATs in Australia, especially in rural and remote areas. Improving access to PD nurses for patients with aPD could help to increase uptake of LD-based DATs in Australia, support treating health care professionals in training patients on use of the pump, provide ongoing follow up support to ensure that patients have the best chance of successful therapy, thus improving QoL for aPD patients and their families, and increasing the social benefit associated with LD-based DATs.

## Discussion

This study is the first published SROI to evaluate the impact of improving access to LD-based DATs for people living with aPD. Results showed that the value of LD-based DATs is experienced not only by patients (27%) and the Government (15%), but that over half of the value is generated for the partners (22%) and children (36%) of people living with aPD. In addition, results highlighted the importance of improvements in non-motor symptoms, such as mental wellbeing, cognition, and speech. These improvements positively impact social and emotional outcomes, including increased hope for the future and increased connection to family and friends. Finally, this research also revealed gaps in aPD care, specifically the need for increased access to specialised nurses with expertise in aPD and LD-based DATs.

Previous studies have evaluated the burden of aPD, including its economic impact and effect on families and carers of people living with aPD (5, 12, 26). Previously published CEAs showed treating aPD with LD-based DATs such as Duodopa® generates a positive return (27, 28). However, these analyses did not consider the broader social impacts of treatment on partners and children of people living with aPD despite the known impact of PD on carer quality of life (4, 5). Noting the limitations with existing research, this study employed an SROI approach which aimed to capture the potential social, economic and environmental impacts of LD-based DATs. Based on the key principles of SROI, this research included extensive stakeholder consultation and outcome mapping, to ensure that all relevant stakeholders and material outcomes were identified and included. This method allows for a more complete measurement of value from a societal perspective and highlights the extensive impact of improved treatment for aPD. Our findings demonstrate over half of the value created by LD-based DATs is experienced by partners and children of people living with aPD. Additionally, this research found the benefits of LD-based DATs extend beyond improvement in motor symptoms. For example, improvements in mental wellbeing, cognition, and speech improve a person’s ability to connect with others, thereby improving connection to family and friends. This then has broader impacts on the person’s partner and children, who experience an increased connection to the person living with aPD. These social and emotional outcomes were found to generate most of the value in this SROI but would typically be excluded from a CEA. Previous research has acknowledged the impact of non-motor symptoms (29), and our findings have identified the additional value created by LD-based DATs for both the partners and children of people living with aPD.

This study has some limitations related to the method and the scope of analysis. Firstly, the SROI method has known limitations (13) which can introduce bias. However, this analysis followed SROI best practice and underwent assurance assessment through Social Value International, increasing the confidence in the results. Secondly, this analysis only considered the impact of LD-based DATs in people living with aPD who reside in the community, excluding people living in out-of-home care such as in residential aged care. This was a pragmatic decision, as it was not considered feasible to engage people living in out-of-home care. People living in aged care are likely to have materially difference experiences compared to those living in the community and aged care costs are a significant contributor to the total economic burden of PD (12). Therefore, further research should be undertaken to better quantify the benefits of LD-based DATs in this setting. Findings could help to inform treatment options for these patients and understand the broader impact of LD-based DATs for this population. Lastly, the avoided cost of healthcare services and utilisation included in this analysis was informed by previous economic evaluations (30). While the cost estimate used in this analysis included hospitalisation, medical services, and allied health, limited data exists detailing the true cost of healthcare services and utilisation related to PD, thus it is likely the savings healthcare utilisation costs are an underestimate. Further, as sensitivity analyses of varying healthcare costs showed a positive return on investment (Supplementary Table S3), this research shows that investing in aPD treatment has social and economic benefits. Future research focusing on the health system cost of PD should be conducted, with a focus on understanding the changing health care resource utilisation as PD progresses. This research will also be useful to inform the broader burden of PD, specifically relating to costs associated with the non-motor symptoms of PD including choking risk and mental health symptoms.

Similarly, the cost of improving access to LD-based DATs for people living with aPD presented in this analysis is based on an estimate of the effective price which may over or underestimate the true price. A sensitivity analysis conducted using the list price still showed a positive return on investment (Supplementary Table S3), demonstrating that even in the most conservative scenario, increased access to LD-based DATs is expected to generate positive social value.

Further research should also assess the potential differential impact of young onset PD. While the average age of PD diagnosis is above 65, approximately 10% of people are diagnosed with PD before the age of 50 (8). People living with young onset PD are more likely to have younger, dependent children, and will likely still be an active part of the workforce. As such, they may experience additional or different outcomes compared to the broader aPD population assessed in this SROI. While some individuals living with young onset PD were consulted as part of this research, additional research focusing exclusively on this population may reveal additional impacts.

## Data availability statement

The datasets presented in this article are not readily available because the original contributions presented in the study are included in the article/Supplementary material. Other raw data supporting the conclusions of this article may be made available upon reasonable request and in line with the requirements of the ethical approval for this research. Further inquiries can be directed to the corresponding author. Requests to access the datasets should be directed to InD, inez.denham@htanalysts.com.au.

## Ethics statement

The studies involving humans were approved by Bellberry Human Research Ethics Committee and the Gold Coast Hospital and Health Service. The studies were conducted in accordance with local legislation and institutional requirements. The participants provided their written informed consent to participate in this study. Written informed consent was obtained from the individual(s) for the publication of any potentially identifiable images or data included in this article.

## Author contributions

InD: Conceptualization, Data curation, Formal analysis, Investigation, Methodology, Project administration, Visualization, Writing – original draft. RM: Conceptualization, Data curation, Formal analysis, Investigation, Methodology, Supervision, Writing – original draft. IrD: Conceptualization, Methodology, Project administration, Supervision, Validation, Writing – review & editing. AM: Conceptualization, Funding acquisition, Writing – review & editing. JBW: Resources, Writing – review & editing. CT: Conceptualization, Supervision, Writing – review & editing.

## References

[1] Poewe W Seppi K Tanner CM Halliday GM Brundin P Volkmann J. Parkinson disease. Nat Rev Dis Primers. (2017) 3:17013. doi: 10.1038/nrdp.2017.13 28332488

[2] Armstrong MJ Okun MS. Diagnosis and treatment of Parkinson disease: a review. JAMA. (2020) 323:548–60. doi: 10.1001/jama.2019.22360 32044947

[3] Sellbach A Silburn P. Management of Parkinson’s disease. Aust Prescr. (2012) 35:183–8. doi: 10.18773/austprescr.2012.084

[4] Henry RS Lageman SK Perrin PB. The relationship between Parkinson's disease symptoms and caregiver quality of life. Rehabil Psychol. (2020) 65:137–44. doi: 10.1037/rep0000313, PMID: 32068420 PMC7195231

[5] Tan SB Williams AF Morris ME. Experiences of caregivers of people with Parkinson's disease in Singapore: a qualitative analysis. J Clin Nurs. (2012) 21:2235–46. doi: 10.1111/j.1365-2702.2012.04146.x, PMID: 22788558

[6] Zhao N Yang Y Zhang L Zhang Q Balbuena L Ungvari GS. Quality of life in Parkinson's disease: a systematic review and meta-analysis of comparative studies. CNS Neurosci Ther. (2021) 27:270–9. doi: 10.1111/cns.13549, PMID: 33372386 PMC7871788

[7] Aldred J Anca-Herschkovitsch M Antonini A Bajenaru O Bergmann L Bourgeois P. Application of the '5-2-1′ screening criteria in advanced Parkinson's disease: interim analysis of DUOGLOBE. Neurodegener Dis Manag. (2020) 10:309–23. doi: 10.2217/nmt-2020-0021, PMID: 32873195

[8] Evans A Fung VSC O'Sullivan JD Stell R White R Williams DR. Characteristics of advanced Parkinson's disease patients seen in movement disorder clinics - Australian results from the cross-sectional OBSERVE study. Clin Park Relat Disord. (2021) 4:100075. doi: 10.1016/j.prdoa.2020.100075, PMID: 34316660 PMC8299979

[9] Schrag A Quinn N. Dyskinesias and motor fluctuations in Parkinson's disease: a community-based study. Brain. (2000) 123:2297–305. doi: 10.1093/brain/123.11.2297 11050029

[10] Calabresi PP Filippo MDMD Ghiglieri VP Tambasco NMD Picconi BP. Levodopa-induced dyskinesias in patients with Parkinson's disease: filling the bench-to-bedside gap. Lancet Neurol. (2010) 9:1106–17. doi: 10.1016/S1474-4422(10)70218-0 20880751

[11] Soileau MJ Aldred J Budur K Fisseha N Fung VSC Jeong A. Safety and efficacy of continuous subcutaneous foslevodopa-foscarbidopa in patients with advanced Parkinson's disease: a randomised, double-blind, active-controlled, phase 3 trial. Lancet Neurol. (2022) 21:1099–109. doi: 10.1016/S1474-4422(22)00400-8, PMID: 36402160

[12] Deloitte Access Economics. (2015). Living with Parkinson's disease–an updated economic analysis 2014. Available at: https://www2.deloitte.com/au/en/pages/economics/articles/living‐with‐parkinsons‐disease.html

[13] Nicholls L Neitzert E Goodspeed T. A guide to Social Return on Investment. London: SROI (2012).

[14] Chaudhuri KR Kovács N Pontieri F Aldred J Bourgeois P Davis T. Long-term motor and non-motor symptom benefits in patients with advanced Parkinson’s disease treated with levodopa-carbidopa intestinal gel: final analysis. Neurology. (2022) 98:2773. doi: 10.1212/WNL.98.18_supplement.2773

[15] Dovetail. (2023) Dovetail. Available at: https://dovetail.com/.

[16] Qualtrics. (2023). Qualtrics. Available at: https://www.qualtrics.com/au/.

[17] Social Value International. (2024). SROI Value Map NR. Available at: https://www.socialvalueint.org/sroi-value-map.

[18] Filippo Montesi FM Nicola C Francesca B. A bridge towards inclusion. Milan, Italy: ExtraBanca (2018).

[19] AbbVie Pty Ltd. M15-736 clinical study report. Australia: AbbVie Pty Ltd (2021).

[20] Pharmaceutical Benefits Advisory Committee (PBAC). (2016). 3A.9 Uncertainty analysis: model inputs and assumptions Available at: https://pbac.pbs.gov.au/section-3a/3a-9-uncertainty-analysis-model-inputs-and-assumptions.html.

[21] Department of Health and Aged Care and The pharmaceutical benefits scheme. (2024). Levodopa + carbidopa. Available at: https://www.pbs.gov.au/medicine/item/1242j

[22] PBS Information Management Section. PBS expenditure and prescriptions report: 1 July 2020 to 30 June 2021. Canberra, Australia: Department of Health (2020).

[23] Independent Health and Aged Care Pricing Authority. (2022). National Hospital Cost Data Collection (NHCDC) public hospitals report - round 24 (financial year 2019–20). Available at: https://www.ihacpa.gov.au/health-care/costing/national-hospital-cost-data-collection

[24] Department of Health and Aged Care. (2023). Medicare benefits schedule - item 105. Available at: https://www9.health.gov.au/mbs/fullDisplay.cfm?type=item&q=105.

[25] Canadian Agency for Drugs and Technologies in Health. (2018). CADTH common drug review: patient input. Levodopa/carbidopa (Duodopa) indication. Available at: https://www.cadth.ca/sites/default/files/cdr/pharmacoeconomic/SR0557_DuodopaFWG_PE_Report.pdf

[26] Hermanowicz N Jones SA Hauser RA. Impact of non-motor symptoms in Parkinson's disease: a PMDAlliance survey. Neuropsychiatr Dis Treat. (2019) 15:2205–12. doi: 10.2147/NDT.S213917, PMID: 31496703 PMC6689087

[27] Chaudhuri KR Pickard AS Alobaidi A Jalundhwala YJ Kandukuri PL Bao Y. The cost effectiveness of levodopa-carbidopa intestinal gel in the treatment of advanced Parkinson’s disease in England. PharmacoEconomics. (2022) 40:559–74. doi: 10.1007/s40273-022-01132-y, PMID: 35307793 PMC9095547

[28] Lowin J Sail K Baj R Jalundhwala YJ Marshall TS Konwea H. The cost-effectiveness of levodopa/carbidopa intestinal gel compared to standard care in advanced Parkinson’s disease. J Med Econ. (2017) 20:1207–15. doi: 10.1080/13696998.2017.1379411, PMID: 28895769

[29] Poortvliet PC Silburn PA Coyne TJ Chenery HJ. Deep brain stimulation for Parkinson disease in Australia: current scientific and clinical status. Intern Med J. (2015) 45:134–9. doi: 10.1111/imj.12656, PMID: 25650534

[30] Bohingamu Mudiyanselage S Watts JJ Abimanyi-Ochom J Lane L Murphy AT Morris ME. Cost of living with Parkinson’s disease over 12 months in Australia: a prospective cohort study. Parkinsons Dis. (2017) 2017:1–13. doi: 10.1155/2017/5932675 PMC5352887 28352490

