# Supplementary material for: Levodopa-based device-aided therapies for the treatment of advanced Parkinson’s disease: a social return on investment analysis
Source: Front Public Health. 2024 Jun 6;12:1351808. doi: 10.3389/fpubh.2024.1351808 (PMC11228815; doi:10.3389/fpubh.2024.1351808)
Supplement: Supplementary file 1 [file Table_1.docx]

Supplementary Material

Levodopa-based device-aided therapies for the treatment of advanced Parkinson’s disease: a Social Return on Investment analysis

Inez Denham^1^*, Roxanne Maurin^1^, Irene Deltetto^1^, Anna Stefanie Mikolaizak^2^, Jenny B. Waern^2^, Colman Taylor^1, 3^

^1^**HT**ANALYSTS Pty Ltd, Sydney, NSW, Australia

^2^AbbVie Pty Ltd, Sydney, NSW, Australia

^3^The George Institute for Global Health, Sydney, NSW, Australia

***Correspondence:**Inez Denham
[inez.denham@htanalysts.com.au](mailto:inez.denham@htanalysts.com.au)

# Supplementary Data

## Stakeholder calculations

Table S1: Stakeholder calculations

| **Stakeholder group** | **Value** | **Rationale** |
| --- | --- | --- |
| **People living with aPD** | | |
| Number of people initiating LD-based DAT per year | AbbVie Pty Ltd commercial-in-confidence assumptions | |
| Total number of people living with aPD receiving treatment with a LD-based DAT | 1,228 | AbbVie Pty Ltd commercial-in-confidence assumptions and data on file |
| **Partners of people living with aPD** | | |
| Proportion of people aged 45+ who are registered as married or de facto | 65% | Proportion of registered married or de facto people in Australia aged 45 and over. In line with Australian population rates, it is assumed 65% of people living with aPD will be married or have a de facto partner. This partner is assumed to be their primary caregiver (1). |
| Total number of partners of people living with aPD | 798 | |
| **Children of people living with aPD** | | |
| Number of children per family | 2 | The most common family size in Australia includes 2 children. It is expected that the average person living with aPD will have 2 children (2). |
| Total number of children of people living with aPD | 2,456 | |
| **Australian Government** | | |
| Total number of Australian Government | 1 | The Australian Federal Government is considered a single stakeholder for the purposes of this analysis. |

Abbreviations: aPD, advanced Parkinson’s disease

## SROI filters and importance

Table S2: SROI filters included in the impact map

| **Stakeholder** | **Outcome(s)** | **Importance** | **Deadweight** | **Attribution** | **Displacement** | **Drop off** |
| --- | --- | --- | --- | --- | --- | --- |
| People living with aPD | Reduced out-of-pocket costs for aids and modifications | 100% | 20% | 0% | 0% | 14% |
|  | Increased independence | 93% | 20% | 0% | 0% | 14% |
|  | Increased connection to family and friends | 81% | 20% | 0% | 0% | 14% |
|  | Increased ability to remain in the workforce | 100% | 20% | 0% | 0% | 20%^b^ |
|  | Increased hope for the future | 73% | 20% | 0% | 0% | 14% |
|  | Increased burden of discomfort | 30% | 0%^a^ | 0% | 0% | 0% |
| Partners of people living with aPD | Increased hope for the future | 88% | 20% | 0% | 0% | 14% |
|  | Reduced worry about partner’s health | 90% | 20% | 0% | 0% | 16%^c^ |
|  | Increased carer wellbeing | 82% | 20% | 0% | 0% | 14% |
|  | Increased connection to family and friends | 80% | 20% | 0% | 0% | 14% |
|  | Increased ability to remain in the workforce | 100% | 20% | 0% | 0% | 20%^b^ |
| Children of people living with aPD | Increased connection to parent | 65% | 20% | 0% | 0% | 14% |
|  | Reduced worry about parent | 90% | 20% | 0% | 0% | 16%^c^ |
| Australian Government | Avoided cost of healthcare services | 100% | 20% | 0% | 0% | 14% |
|  | Avoided cost of welfare services and support payments | 100% | 20% | 0% | 0% | 20%^b^ |

Abbreviations: aPD, advanced Parkinson’s disease

^a^ Discomfort with the infusion site, pump, and tube is entirely due to treatment with a levodopa-based DAT. No other factor contributes to this outcome.
^b^ 27% of people in the 45-64 age group who are currently working full-time intend to retire in the next five years. This rate has been added to the discontinuation rate to account for people organically leaving the workforce.
^c^ An estimated 1.5% of people aged 65 years and above will have an admission to permanent residential aged care each year. This rate has been added to the discontinuation rate to account for people transitioning to permanent residential aged care based exclusively on age.

## Sensitivity analyses

Table S3: Sensitivity analysis results

| **Type** | **Scenario** | **Value** | **SROI ratio, NPV** | **% change in SROI ratio** |
| --- | --- | --- | --- | --- |
| Discount rate | Base case | 5.00% | 1:1.83  $187.80 million | - |
|  | Alternative discount rate suggested in PBAC guidelines | 3.50% | 1:1.82  $189.80 million | +1.10% |
|  | Alternative discount rate suggested in PBAC guidelines | 5.00% | 1:1.82  $194.74 million | +3.70% |
| Cost input | Base case | $49,446 per person living with aPD  ($60.72 million total annual investment) | 1:1.83  $187.80 million | - |
|  | Nil Special Pricing Agreement | $83,324.00 per person living with aPD  ($102.3 million total annual investment) | 1:1.42  $122.38 million | -22.00% |
|  | Breakeven: Cost of levodopa-based DATs for NPV to equal $0 (SROI ratio 1:1) | $117,554 per person living with aPD  ($144.4 million total annual investment) | 1:1  $0.00 | -83.00% |
| Valuation approaches and financial proxies – increased independence for people living with aPD | Base case | $53,268.10 | 1:1.83  $187.80 million | - |
|  | Replacement valuation - Lower Care Needs for Home Care Package (Level 3) | $35,138.55 | 1:1.71  $162.27 million | -6.20% |
| Valuation approaches and financial proxies – increased carer wellbeing for partners of people living with aPD | Base case | $22,330.35 | 1:1.83  $187.80 million | - |
|  | Replacement valuation - average care of aged care homes to reflect the value of increased carer wellbeing | $48,535.77 | 1:1.93  $211.34 million | +5.70% |
| Valuation approaches and financial proxies – increased connection to parent for children of people living with aPD | Base case | $972.00 | 1:1.83  $187.80 million | - |
|  | Replacement valuation – average cost of family therapy | $2,411.47 | 1:1.84  $190.64 million | +0.70% |
|  | Replacement valuation – international flight reflects the cost of connection to parent | $1,400.00 | 1:1.83  $188.65 million | +0.20% |
| Valuation approaches and financial proxies – avoided cost of healthcare services for the Australian Government | Base case | $30,806.28 | 1:1.83  $187.80 million | - |
|  | Alternative source of health system cost of PD care resource utilisation | $27,434.97 | 1:1.80  $182.12 million | -1.40% |
| Time horizon and duration | Base case | 3 years^a^ | 1:1.83  $187.80 million | - |
|  | One year time horizon | 1 year | 1:2.12  $88.96 million | +16.00% |
|  | 5 year time horizon | 5 years^a^ | 1:1.59  $214.39 million | -13.00% |
| Attribution | Base case | 0.00% | 1:1.83  $187.80 million | - |
|  | Increased attribution from base case – the outcomes included in this SROI were almost entirely the result of access to levodopa-based DATs | +20.00% for each outcome^b^ | 1:1.46  $104.65 million | -20.00% |
|  | Increased attribution from base case – the outcomes included in this SROI were largely the result of access to levodopa-based DATs | +40.00% for each outcome^b^ | 1:1.09  $21.50 million | -40.00% |
| Deadweight | Base case | 20.00% | 1:1.83  $187.80 million | - |
|  | Decreased deadweight from base case – the outcomes included in this SROI would never have occurred without access to levodopa-based DATs | -20.00% for each outcome^c^ | 1:2.28  $291.75 million | +25.00% |
|  | Increased deadweight from base case – the outcomes included in this SROI might have occurred without access to treatment with levodopa-based DATs | +20.00% for each outcome^c^ | 1:1.37  $83.87 million | -25.00% |
| Displacement | Base case | 0.00% | 1:1.83  $187.80 million | - |
|  | Increased displacement from base case | +20.00% for each outcome | 1:1.46  $104.65 million | -20.00% |
|  | Increased displacement from base case | +40.00% for each outcome | 1:1.09  $21.50 million | -40.00% |

N.B. rounding applies

Abbreviations: aPD, advanced Parkinson’s disease; DAT, device-assisted therapy; NPV, net present value; PBAC, Pharmaceutical Benefits Advisory Committee; PD, Parkinson’s disease; SROI, Social Return on Investment

^a^ Discomfort associated with the infusion site, pump, and tube was found to be temporary. Thus, the duration of this outcome is one year.

^b^ Discomfort associated with the infusion site, pump, and tube is a direct result of initiating treatment with levodopa-based DATs. Thus, attribution for this outcome was 0%.

^c^ Discomfort with the infusion site, pump, and tube is entirely due to treatment with a levodopa-based DAT. No other factor contributes to this outcome. Thus, deadweight for this outcome was 0%.

# References

1. Australian Bureau of Statistics (ABS). Household and families: Census. 2021.

2. Australian Government | Australian Institute of Family Studies. Families then & now: having children. 2020.
